# Oral Health in Iraqi Schoolchildren: A Comprehensive Cross-Sectional Analysis of Sociodemographic Factors, Behavioural Patterns, and Parental Knowledge Influencing Dental Caries

**DOI:** 10.3290/j.ohpd.c_2027

**Published:** 2025-06-03

**Authors:** Hanan Fadhil Alautry, Mohammad Hossein Khoshnevisan, Mahshid Namdari, Hadi Ghasemi

**Affiliations:** a Hanan Fadhil Alautry Assistant Professor, Department of Community Oral Health, School of Dentistry, Shahid Beheshti University of Medical Sciences, Tehran, Iran; Department of Pediatric and Preventive Dentistry, School of Dentistry, Wasit University, Wasit, Iraq. Methodology, formal analysis, collected the data, drafted the manuscript, performed statistical analyses, resources, revised the final manuscript.; b Mohammad Hossein Khoshnevisan Professor, Department of Community Oral Health, School of Dentistry, Shahid Beheshti University of Medical Sciences, Tehran, Iran. Supervision, revised the final manuscript.; c Mahshid Namdari Associate Professor, Department of Biostatistics, School of Allied Medical Sciences, Shahid Beheshti University of Medical Sciences, Tehran, Iran. Methodology, statistical analysis, revised the final manuscript.; d Hadi Ghasemi Associate Professor, Head of Department of Community Oral Health, School of Dentistry, Shahid Beheshti University of Medical Sciences, Tehran, Iran. Methodology, supervision, drafted the manuscript, revised the final manuscript.

**Keywords:** dental caries, parental knowledge, oral health behaviours, schoolchildren, sociodemographic factors.

## Abstract

**Purpose:**

To evaluate the caries status and its associated factors among Iraqi schoolchildren.

**Materials and Methods:**

A cross-sectional study was conducted from October to December 2022 with 372 primary schoolchildren aged 8–10 years in Kut City, Iraq. The study participants were selected using a multi-stage random sampling technique. Information about the children was collected through a questionnaire that included demographic characteristics, oral health-related behaviours, and parental knowledge regarding oral health. Moreover, a clinical dental examination was performed, which included assessment of decayed, missing, and filled teeth (DMFT, dmft) based on the criteria of the World Health Organization (WHO). The statistical analysis included the chi-squared test, ANOVA, and simple and multiple logistic regressions.

**Results:**

The children’s mean age was 9.0 years (± 0.82). The overall caries prevalence among the children was 94%. In terms of caries experience, in the primary dentition, 84% of the children had a mean dmft = 4, and in the permanent dentition, 61% of the children had a mean DMFT = 1.5. Multiple logistic regression showed that lower maternal educational level (OR = 2.10, 95% CI: 0.43–10.07), no history of dental visits (OR = 10.99, 95% CI: 2.29–52.72), and poor parental knowledge (OR = 7.70, 95% CI: 1.74–34.12) were positively associated with the prevalence of untreated tooth decay in this group of schoolchildren.

**Conclusion:**

Dental caries was found to be highly prevalent, while a favourable level of oral health behaviours was rare among schoolchildren in this study. The mother’s educational level, parents’ knowledge about oral health, and having a dental visit in the last year were found to be associated with caries.

Dental caries continues to impact the well-being of children worldwide, reflecting a substantial public health concern.^17^ It stands out as one of the most frequent childhood diseases, contributing significantly to the global health burden.^35^ The consequences of untreated caries extend beyond oral health, becoming a primary source of pain and negatively influencing a child’s dietary habits and sleep quality.^26^ According to the World Health Organization (WHO), caries affects a substantial proportion of schoolchildren, ranging from 60% to 90% worldwide.^40^

The multifaceted nature of caries is evident, with varying disease levels observed both among countries and within specific regions. These variations are attributed to diverse factors such as living conditions, environmental elements, lifestyle choices, and the accessibility of preventive resources.^15^

As caries can adversely affect a child’s overall health and academic performance, a comprehensive examination of contributing factors become imperative. Given the importance of oral health in children, investigations on the prevalence of caries and its associated factors among schoolchildren have been conducted in various regions of the world. In this regard, varying prevalences of caries among primary school pupils have been reported, for example, 83% in Saudia Arabia,^3^ 70% in Italy,^10^ 56% in China,^9^ 44% in Japan,^29^ and 19% in the USA.^28^ In addition, several studies have investigated factors that correlate with the development of caries in schoolchildren, such as the child’s age^18,27^ and oral health behaviours,^22,33^ parental educational attainment^10,12,13^ awareness about caries prevention.^19,34^

In Iraq, with approximately 4,864,000 primary schoolchildren^37^ and an average DMFT of 1.5 (±1.59),^2^ little is known about the status and determinants of caries among primary schoolchildren. Caries prevalence among Iraqi schoolchildren has been reported to be 61%,^2,20^ and one of these previous studies in Iraq found an association between maternal educational level and caries.^2^

Therefore, this study seeks to explore caries status and associated factors among schoolchildren aged 8-10 years in the city of Kut, Iraq. By exploring the multifaceted aspects of this oral health challenge, we endeavor to provide valuable information that can be used for targeted interventions, policy development, and educational initiatives to promote better oral health outcomes for Iraqi primary schoolchildren. The findings would serve as a foundation for devising relevant strategies and implementing intervention programs to manage caries and enhance oral health among these schoolchildren in the future.

## MATERIALS AND METHODS

This cross-sectional study targeted children aged 8-10 from elementary schools in Kut City, Iraq. Multistage random sampling was performed. The sample size was determined using G*power version 3.1.9.7,^16^ aiming for 17 predictors in the regression model with an anticipated effect size of 0.15,^34^ a power of 0.90, and α = 0.05, requiring 179 samples. Accounting for a design effect of 1.6 due to cluster sampling, 310 subjects were needed. To mitigate potential drop-out due to incomplete responses, an extra 20% was added, resulting in a final sample of 372 pupils. The distribution ensured equal representation across gender and age, with 62 children from each age group (8, 9, and 10 years), including an equal number of girls (31) and boys (31). Inclusion criteria stipulated Iraqi nationality and an age range of 8-10 years, with strict adherence to exclusion criteria (systemic illness, disabilities, or failure to provide consent). Two girls’ and two boys’ schools were randomly selected via lottery from a list of 40 elementary schools in the city to reach the study subjects.

The questionnaires employed in this study encompassed three distinct sections. The first section gathered demographic information about the students, including age, gender, parents’ age and level of education. The second section focused on aspects of the students’ oral health-related behaviour, examining variables such brushing frequency, consumption of sugary snacks, and the timing of their last dental visit. The third section drew from questionnaires used in two prior studies^6,18^ and included 20 statements pertaining to parental knowledge about oral health and caries preventive measures.

In this segment, parents were asked to express their views on each statement using a 3-point Likert scale (disagree, do not know, and agree), with corresponding scores of 0, 1, and 2. The cumulative scores, theoretically ranging from 0 to 40, constituted the total knowledge score for each respondent, with higher scores indicative of a higher level of knowledge. To simplify this variable, the median of the final scores served as the dividing point. Respondents scoring below the median were categorised as having poor knowledge, while those scoring at or above the median were classified as possessing good knowledge, which follows the approach outlined in a previous study.^18^

To ensure the validity and reliability of the parental knowledge questionnaire, several measures were implemented. Initially, the English version was translated into Arabic using the forwards-backwards technique.^5^ Subsequently, the content validity index (CVI) and content validity ratio (CVR) were computed for all items. Eight experts from the Departments of Pediatric Dentistry and Dental Public Health assessed the necessity, simplicity, clarity, and relevance of each item, resulting in a CVI of 0.94 and CVR of 0.92, indicating an acceptable level of validity.^25,42^ As a third step, the reliability of this questionnaire section was evaluated, in which parents of 20 schoolchildren were enlisted to complete the questionnaire twice, with a two-week interval between administrations, utilising the test-retest method.^30^ The analysis of this process yielded an intraclass correlation coefficient (ICC) of 0.83, which fell within the acceptable range for reliability assessment.

This study received approval from the Ethics Committee of the Shahid Beheshti School of Dentistry (IR.SBMU.DRC.REC.1401.030). Additionally, permission was obtained from the General Directorate of Education of Kut City and the respective school principals. The study’s objectives were communicated to students in each class by one of the authors (HA). Subsequently, the questionnaire and written consent form were distributed among the students to take home. Students were instructed to pass the questionnaire and consent form to their parents for completion, signing, and subsequent return to the schools in the following days. Any inquiries regarding the questionnaire’s content were addressed by HA, providing clarification to both children and their parents. Data collection also involved a clinical examination adhering to the methods recommended by the World Health Organization (WHO).^39^ This evaluation involved documenting the presence of decayed, missing, and filled teeth, denoted as dmft/DMFT. An experienced dentist (HA) conducted the clinical oral examination. Prior to the primary data collection, a calibration program was implemented, wherein the examiner assessed caries experience in a group of 20 children (10 boys and 10 girls) selected from designated schools twice, with a two-week interval. Importantly, the data obtained from these children were not integrated into the main study. The kappa coefficient for intra-examiner agreement exceeded 0.8, indicating acceptability.^36^

Throughout the recruitment period (October to December 2022), on each working day, a group of five children was brought to the examination room facilitated by the school authority. After verifying signed consent and completed questionnaires, the oral examination was conducted using WHO probes (Dentirak; Dearborn Heights, MI, USA) featuring a 0.5-mm ball end. Disposable mouth mirrors were employed, and the examination took place with the child seated on a mobile dental chair, using dental-unit light.

ANOVA was used to determine variations in the mean dt/DT concerning categorical variables. Simple logistic regression analysis was conducted to identify potential associated factors, and variables with a statistical significance level of p < 0.05 were subsequently incorporated into a multiple logistic regression model. This multiple logistic regression model aimed to ascertain the likelihood of variables being correlated with the students’ caries status, treated as a binary variable where 0 indicated no untreated decay and 1 denoted the presence of untreated decay. All statistical analyses adhered to a statistical significance threshold of p < 0.05. The IBM SPSS program version 25 (Armonk, NY, USA) was employed for data analysis.

## RESULTS

As shown in Table 1, the research included 372 children, with a well-distributed age representation, mothers predominantly under the age of 40, and a prevalence of highly educated parents. Daily toothbrushing was reported by most participants, while eating sweet snacks between meals twice or more per day was common, and a substantial proportion had not attended prior dental visits. The prevalence of good parental knowledge regarding preventive oral health measures was nearly equivalent between genders, although girls with more highly educated mothers brushed their teeth statistically significantly more often than did boys.

**Table 1:** Distribution of the children (n = 372) according to their sociodemographic factors and oral health behaviours

Variables	All n (%)	Boys n (%)	Girls n (%)	p-value*
Age (years)	8	122 (33)	58 (30)	64 (35)	0.237
9	119 (32)	58 (30)	61 (34)
10	131 (35)	75 (40)	56 (31)
Mother’s age (years)	< 40	242 (65)	119 (62)	123 (68)	0.253
≥ 40	130 (35)	72 (38)	58 (32)
Father’s education level	Primary school	49 (13)	28 (15)	21 (12)	0.685
Secondary or high school	123 (33)	63 (33)	60 (33)
College graduate	200 (54)	100 (52)	100 (55)
Mother’s educational level	Primary school	58 (16)	41(22)	17 (9)	0.001
Secondary or high school	128 (34)	53 (28)	75 (41)
College graduate	186 (50)	97 (50)	89 ((50)
Brushing frequency	Irregular	54 (15)	31 (16)	23(13)	0.002
Once a day	199 (53)	115 (60)	84 (47)
≥ Twice per day	119 (32)	45 (24)	74 (40)
Sweet snacks between meals	< Twice per day	132 (36)	71 (37)	61 (34)	0.484
≥ Twice per day	240 (64)	120 (63)	120 (66)
Past dental visits	Never	152 (41)	69 (36)	83 (46)	0.052
Last year and before	140 (38)	83 (44)	57 (32)
During the past year	80 (21)	39 (20)	41 (22)
Parental knowledge	Poor	155 (42)	77 (40)	78 (43)	0.587
Good	217 (58)	114 (60)	103 (57)
*Chi-squared test.

Table 2 delineates the caries status within the study group, revealing dental decay percentages of 84.1% and 61.3% in primary and permanent dentition, respectively. The overall prevalence of caries stood at 93.8%, with only 6.2% of children being caries-free. Notably, the decayed component constituted a major proportion of the indices in both dentitions.

**Table 2:** Caries status in the schoolchildren (n = 372)

	Number (%) of children with caries	dt/DT Mean (SD)	mt/MT Mean (SD)	ft/FT Mean (SD)	dmft/DMFT Mean (SD)
Primary teeth	313 (84.1)	3.6 (2.78)	0.35 (0.82)	0.08 (0.48)	4.0 (2.95)
Permanent teeth	228 (61.3)	1.4 (1.56)	0.04 (0.10)	0.06 (0.27)	1.5 (1.6)
Overall prevalence	349 (93.8)	5.0 (3.13)	0.3 (0.83)	0.1 (0.50)	5.4 (5.55)
DT/dt = decayed teeth, MT/mt = missing teeth, FT/ft = filled teeth.

**Fig 1:**
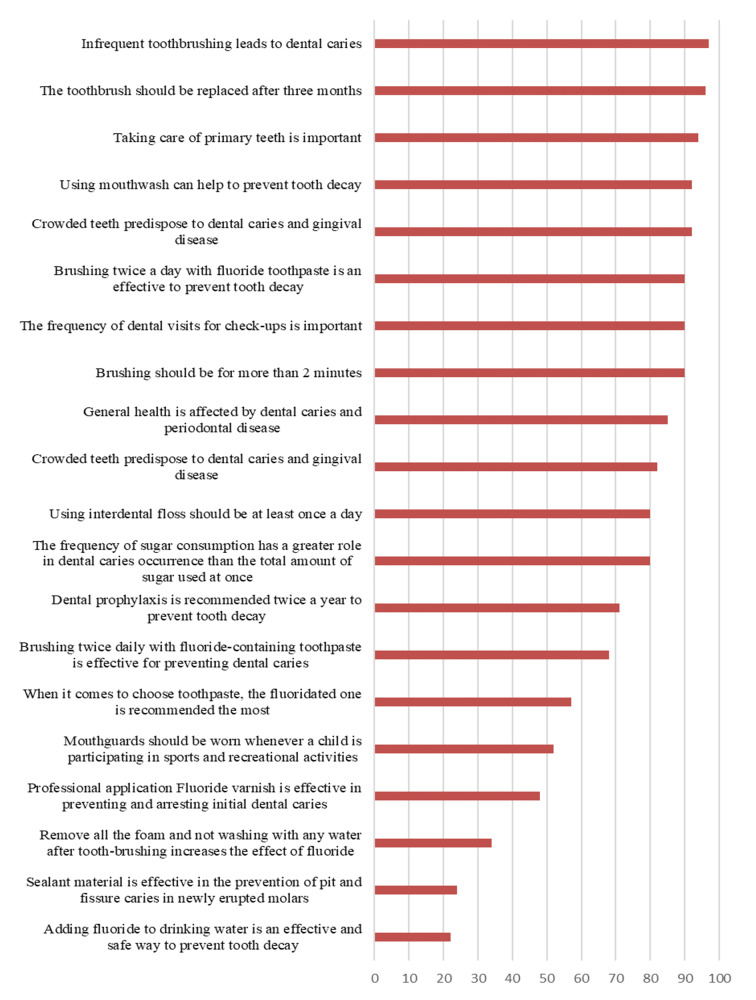
Distribution (%) of children’s parents who agreed with each of the statements about oral health preventive measures.

Figure 1 illustrates parental agreement percentages on various oral health knowledge statements, with over half of the parents falling into the category of possessing good knowledge.

Table 3 outlines the associations between caries and sociodemographic factors, oral health behaviours, and parental knowledge. Lower dt/DT mean scores were correlated with a higher frequency of toothbrushing, more frequent past dental visits, higher parental knowledge scores, and a lower frequency of sweet snacking among the children (p<0.001).

**Table 3:** Relationship between mean dt/DT and selected variables

Variables	Mean (SD) dt/DT	p-value*
Gender	Girls	5 (3.00)	0.895
Boys	5 (3.26)
Mother’s age (years)	< 40 years	4.9 (3.11)	0.581
≥ 40 years	5.1 (3.17)
Father’s educational level	Primary school	5.3 (3.22)	0.375
Secondary or high school	5.1 (3.12)
College graduate	4.8 (3.07)
Mother’s educational level	Primary school	5.5 (3.39)	0.095
Secondary or high school	5.3 (2.85)
College graduate	4.7 (3.21)
Brushing frequency	Irregular	6.5 (2.50)	<0.001*
Once a day	4.5 (2.80)
≥ Twice per day	4.2 (3.17)
Sweet snacks between meals	< Twice per day	4.1 (3.11)	<0.001*
≥ Twice per day	5.8 (3.02)
Past dental visits	During the last six months	3.8 (2.82)	<0.001*
Last year and before	5.3(3.00)
Never	5.8 (3.07)
Parental knowledge	Poor	6 (3.18)	<0.001*
Good	4.3 (2.89)
* ANOVA.

Table 4 identifies three variables as potential associated factors of untreated decay (dt/DT); these were entered into a multiple logistic regression model. The probability of untreated caries was higher for children with mothers who had a primary-school education (OR = 2.10, 95% CI: 0.43 to 10.07), those with past dental visits in the last year or no history of dental visits (OR = 2.96, 95% CI: 1.14 to 7.67; OR = 10.99, 95% CI: 2.29 to 52.72), and those with poor parental knowledge about oral health (OR = 7.70, 95% CI: 1.74 to 34.12).

**Table 4:** Factors associated with having untreated decay (0 = dt/DT = 0, 1 = dt/DT>0) among Iraqi schoolchildren (n = 372) as assessed by simple and multiple binary logistic regression models

Variables	Simple binary logistic models	Multiple binary logistic model
Crude OR	95% CI	p-value*	Adj OR**	95% CI	p-value*
Gender (reference: girls)	1.26	(0.55, 2.90)	0.577			
Mother’s age (reference < 40 years)	1.32	(0.53, 3,28)	0.540			
Father’s education (reference: college graduate)
Primary school	2.04	(0.45, 9.19)	0.352			
Secondary/high school	1.69	(0.64, 4.45)	0.284			
Mother’s education (reference: college graduate)
Primary school	4.74	(1.37, 16.37)	0.014*	2.10	(0.43, 10.07)	0.034*
Secondary/high school	3.18	(0.71, 14.10)	0.127	4.00	(1.11, 14.39)	0.352
Brushing frequency (reference: twice a day)
Once a day	0.71	(0.30, 1.66)	0.432	0.635	(0.25, 1.60)	0.436
Never	4.04	(0.51, 31.78)	0.184	2.32	(0.27, 19.39)	0.336
Sweet snacks (reference: <once a day)
Once a day	0.60	(0.12, 2.84)	0.523			
≥ Twice per day	2.00	(0.40, 9.77)	0.394			
Past dental visits (reference: in six months)
Last year and before	3.80	(1.55, 9.28)	0.003*	2.96	(1.14, 7.67)	0.025*
Never	13.66	(2.95, 63.16)	0.001*	10.99	(2.29, 52.72)	0.003*
Parental knowledge (reference: good )	8.63	(1.99, 37.29)	0.004*	7.70	(1.74, 34.12)	0.007*
Note: those variables with p>0.25 in the simple logistic regression model were not included in the multiple logistic regression model, which resulted in some empty cells in the table.

## DISCUSSION

The current investigation presents the most recent update on the prevalence of caries in 8–10-year-old schoolchildren in the Kut City, Iraq. The study reveals a notably high overall prevalence of caries, reaching about 94% among the primary-school-age population. Furthermore, untreated decay is found to be linked to various factors such as oral health behaviours of children, maternal educational level, and parental knowledge. Consistent with numerous research reports spanning the past decades,^1,21,31^ this study underscores the well-established association between caries and social as well as behavioural factors.

In this investigation, the prevalence of caries and the mean dmft for deciduous teeth among the studied schoolchildren were considerably higher at 84.1% and 4.1, respectively, compared to recent similar studies in China (39%, 1.6)^8^ and Libya (71%, 2.3).^4^ This disparity may be attributed to a potential underestimation by parents of the importance of deciduous teeth,^38^ leading to limited access to or insufficient utilisation of dental services.

The caries prevalence for permanent teeth among the participating schoolchildren in this study (61%) was very similar to previous findings among Iraqi schoolchildren (64%),^27^ and the Eastern Mediterranean Region (66%),^23^ yet higher than rates reported in Iran (41%),^41^ Italy (37%),^11^ and China (21%).^8^ This highlights the need for future oral health policies in Iraq to prioritise caries-preventive measures and caries-associated complications among primary schoolchildren.

Findings of the present study align with earlier investigations in Iran^21^ and Lao People’s Democratic Republic,^32^ where caries prevalence demonstrated an inverse relationship with the frequency of toothbrushing. Our study corroborated this trend and additionally found a correlation between caries status and the frequency of sweet snack consumption, a connection supported by prior research in China^8^ and Japan.^24^ The rapid economic development and increased exposure to foreign diets have led to schoolchildren having more access to sweet foods and soft drinks.^43^ Although a majority of parents in the current study acknowledged the important role of sugar consumption frequency in the occurrence of caries, nearly two-thirds of schoolchildren still indulged in sweet snacks at least twice per day.

This study identified three variables that contribute to caries among primary schoolchildren: past dental visits, parents’ educational level and oral health knowledge.

A statistically significant association was found between untreated caries and past dental visits, corroborating the findings of a similar study conducted in China.^22^

Additionally, the present study confirmed a statistically significant link between parents’ educational level and untreated caries. This is in line with earlier reports from Italy^1^ and Egypt,^10^ where higher parental education was associated with improved oral hygiene practices among children, including more frequent toothbrushing, dental visits, and regular check-ups.^7^ Parents with a higher educational level may have better oral health awareness or higher income and thus better living and health conditions than other parents, which in turn leads to caries reduction in their children.^14^ Efforts directed at improving socioeconomic status should be continued, owing to evidence from the present study and prior studies that identified maternal education as a factor consistently associated with caries.

Our results demonstrated a statistically significant disparity in the mean dmft/DMFT among children based on their parents’ levels of knowledge on oral health, consistent with findings from previous studies in China,^8^ Croatia,^19^ and Finland.^33^ Furthermore, this variable was identified as a factor contributing to caries. These outcomes underscore the crucial role that mothers play in promoting the oral health of their children and emphasise the important of involving parents in school-based oral health education programs to yield lasting benefits.

One limitation of this study is the cross-sectional study design, in which no causal association between caries and its determinants could be established. In addition, inherent bias (over-reporting of favourable behaviours related to oral hygiene practices) can be expected. However, the respondents were asked to be honest in their responses and were ensured there would be no negative consequences based on their answers. Another limitation of the present study was that some important variables were not included, e.g., family income, parents’ careers, schoolchildren’s oral health knowledge and attitudes, etc. On the other hand, this study has several strengths. Notably, it represents an initial endeavor to assess the relationship between caries status and associated factors among children aged 8-10 years in Kut City, Iraq. The application of random sampling techniques ensured the selected children’s representativeness, enhancing the findings’ generalisability for all schoolchildren in Kut City, Iraq.

## CONCLUSION

The high prevalence of caries, particularly in the primary dentition, along with a suboptimal level of oral health behaviours among schoolchildren in this investigation underscores the urgent need for effective preventive measures. The educational level of the mother, the parents’ knowledge about oral health, and having regular dental visits were found to be associated with caries. These findings stress the need for targeted interventions that address sociodemographic factors, promote oral health behaviours, and enhance parental knowledge in order to alleviate the burden of caries in this particular population. These essential steps are expected to improve children’s oral health and reduce the burden of untreated caries in the community.

## ACKNOWLEDGEMENTS

The authors acknowledge and thank the General Directorate of Education in Kut City for their help. We also thank the participating schools’ directors, teachers, and pupils for their cooperation in implementing this study.

## References

[ref1] Abbass MMS, Mahmoud SA, El Moshy S, Rady D, AbuBakr N, Radwan IA (2019). The prevalence of dental caries among Egyptian children and adolescences and its association with age, socioeconomic status, dietary habits and other risk factors. A cross-sectional study. F1000Res.

[ref2] Alautry HF, Namdari M, Khoshnevisan MH, Ghasemi H (2024). Association between dental clinical measures and oral health-related quality of life among Iraqi schoolchildren: A cross-sectional study. PLoS ONE.

[ref3] Alhabdan YA, Albeshr AG, Yenugadhati N, Jradi H (2018). Prevalence of dental caries and associated factors among primary school children: a population-based cross-sectional study in Riyadh, Saudi Arabia. Environ Health Prev Med.

[ref4] Ballo L, Arheiam A, Marhazlinda J (2021). Determinants of caries experience and the impact on the OHRQOL of 6-year-old Libyan children: a cross-sectional survey. BMC Oral Health.

[ref8] Chen Z, Zhu J, Zhao J, Sun Z, Zhu B, Lu H, Zheng Y (2023). Dental caries status and its associated factors among schoolchildren aged 6-8 years in Hangzhou, China: a cross-sectional study. BMC Oral Health.

[ref9] Cheng YH, Liao Y, Chen DY, Wang Y, Wu Y (2019). Prevalence of dental caries and its association with body mass index among school-age children in Shenzhen, China. BMC Oral Health.

[ref10] Cianetti S, Lombardo G, Lupatelli E, Rossi G, Abraha I, Pagano S (2017). Dental caries, parents educational level, family income and dental service attendance among children in Italy. Eur J Paediatr Dent.

[ref12] Crocombe LA, Allen P, Bettiol S, Babo Soares LF (2018). Parental education level and dental caries in school children living in Dili, Timor-Leste. Asia Pac J Public Health.

[ref14] Du MQ, Li Z, Jiang H, Wang X, Feng XP, Hu Y (2018). Dental caries status and its associated factors among 3- to 5-year-old children in China: a national survey. Chin J Dent Res.

[ref16] Faul F, Erdfelder E, Buchner A, Lang AG (2009). Statistical power analyses using G*Power 3.1: tests for correlation and regression analyses. Behav Res Methods.

[ref18] Folayan MO, Kolawole KA, Oyedele T, Chukwumah NM, Onyejaka N, Agbaje H (2014). Association between knowledge of caries preventive practices, preventive oral health habits of parents and children and caries experience in children resident in sub-urban Nigeria. BMC Oral Health.

[ref19] Gavic L, Tadin A, Matkovic A, Gorseta K, Sidhu SK (2022). The association of parental dental anxiety and knowledge of caries preventive measures with psychological profiles and children’s oral health. Eur J Paediatr Dent.

[ref20] Ghasemi H, Alautry HF, Khoshnevisan MH, Namdari M (2025). Effectiveness of a school-based oral health promotion program on dental caries among Iraqi school children: a cluster randomised controlled trial. Int Dent J.

[ref21] Ghasemianpour M, Bakhshandeh S, Shirvani A, Emadi N, Samadzadeh H, Moosavi Fatemi N (2019). Dental caries experience and socio-economic status among Iranian children: a multilevel analysis. BMC Public Health.

[ref22] Hu J, Jiang W, Lin X, Zhu H, Zhou N, Chen Y, Wu W (2018). Dental caries status and caries risk factors in students ages 12-14 years in Zhejiang, China. Med Sci Monit.

[ref23] Kale S, Kakodkar P, Shetiya S, Abdulkader R (2020). Prevalence of dental caries among children aged 5–15 years from 9 countries in the Eastern Mediterranean Region: a meta-analysis. East Mediterr Health J.

[ref24] Kato H, Tanaka K, Shimizu K, Nagata C, Furukawa S, Arakawa M (2017). Parental occupations, educational levels, and income and prevalence of dental caries in 3-year-old Japanese children. Environ Health Prev Med.

[ref25] Lawshe CH (1975). A quantitative approach to content validity. Personnel Psych.

[ref26] Lima SLA, Santana CCP, Paschoal MAB, Paiva SM, Ferreira MC (2018). Impact of untreated dental caries on the quality of life of Brazilian children: population-based study. Int J Paediatr Dent.

[ref27] Moca AE, Vaida LL, Negruțiu BM, Moca RT, Todor BI (2021). The influence of age on the development of dental caries in children. A radiographic study. J Clin Med.

[ref28] Mohajeri A, Berg G, Watts A, Cheever VJ, Hung H (2024). Obesity and dental caries in school children. J Clin Med.

[ref31] Peres MA, de Oliveira Latorre Mdo R, Sheiham A, Peres KG, Barros FC, Hernandez PG (2005). Social and biological early life influences on severity of dental caries in children aged 6 years. Community Dent Oral Epidemiol.

[ref32] Phanthavong S, Nonaka D, Phonaphone T, Kanda K, Sombouaphan P, Wake N (2019). Oral health behavior of children and guardians’ beliefs about children’s dental caries in Vientiane, Lao People’s Democratic Republic (Lao PDR). PLoS One.

[ref33] Poutanen R, Lahti S, Tolvanen M, Hausen H (2006). Parental influence on children’s oral health-related behavior. Acta Odontol Scand.

[ref34] Quadri MFA, Alwadani MA, Talbi KM, Hazzazi RAA, Eshaq RHA, Alabdali FHJ (2022). Exploring associations between oral health measures and oral health-impacted daily performances in 12-14-year-old schoolchildren. BMC Oral Health.

[ref35] Selwitz RH, Ismail AI, Pitts NB (2007). Dental caries. Lancet.

[ref38] Vittoba Setty J, Srinivasan I (2016). Knowledge and awareness of primary teeth and their importance among parents in Bengaluru City, India. Int J Clin Pediatr Dent.

[ref42] Zamanzadeh V, Ghahramanian A, Rassouli M, Abbaszadeh A, Alavi-Majd H, Nikanfar AR (2015). Design and implementation content validity study: development of an instrument for measuring patient-centered communication. J Caring Sci.

[ref43] Zhen S, Ma Y, Zhao Z, Yang X, Wen D (2018). Dietary pattern is associated with obesity in Chinese children and adolescents: data from China Health and Nutrition Survey (CHNS). Nutr J.

